# For Dandruff

**Published:** 1889-09

**Authors:** 


### FOR DANDRUFF.

Take borax, half a teaspoonful; common sulphur, one heaping teaspoonful; pour over this one pint of boiling water. When cool, pour into a bottle; agitate frequently for three or four days, then strain. Moisten the scalp thoroughly with this two or three times a week. It is one of the most, if not the most reliable preparations known for permanently removing dandruff.

### SHAMPOO LIQUID.

For cleansing the head from dandruff:

| | |
|---|---|
| R Carbonate of Ammonia | 1 drachm. |
| Carbonate of Potassium | 1 drachm. |
| Water | 4 oz. |
| Tinct. of Cantharis | 1 drachm. |
| Alcohol | 4 oz. |
| Rum | 1½ pints. |

Dissolve the carbonates in the water; shake well before using, moistening scalp well with this till a lather forms. Wash in cool water and rub.

